# Chemokine Expression in Human Astrocytes in Response to Shiga Toxin 2

**DOI:** 10.1155/2012/135803

**Published:** 2012-12-10

**Authors:** Naomi Kioka, Koichi Minami, Akira Tamura, Norishige Yoshikawa

**Affiliations:** Department of Pediatrics, Wakayama Medical University School of Medicine, 811-1 Kimiidera, Wakayama 641-0012, Japan

## Abstract

Infection with Shiga toxin- (Stx-) producing *Escherichia coli* can lead to hemolytic uremic syndrome (HUS). Approximately, 30% of patients with HUS suffer from complications in the central nervous system (CNS), which is an important determinant of mortality in such patients. Autopsy shows mostly edema and hypoxic-ischemic changes in the CNS, often with microhemorrhages. It has been suggested that Stx-induced damage to human brain endothelial cells, which are essential constituents of the blood-brain barrier, plays a crucial role in the development of the CNS complications. However, it is unclear whether Stx affects brain neuroglial cells. In the present study, we investigated the direct involvement of Stx in the inflammatory responses of human astrocytes (HASTs) treated with Stx. Immunohistochemistry and real-time PCR revealed that the expression of globotriaosylceramide (Gb3), the receptor for Stx2, and Gb3 synthase (GalT6) in HASTs was increased by interleukin-1**β** (IL-1**β**). Expression of both interleukin-8 (IL-8) and monocyte chemoattractant protein-1 (MCP-1) mRNA in HASTs was significantly upregulated by Stx2. These results suggest that Stx2 induces inflammatory responses, particularly through expression of chemokines, in HASTs expressing Gb3 and may, thus, affect brain glial cells, playing a key role in the pathogenesis of CNS manifestations associated with HUS.

## 1. Introduction

Hemolytic uremic syndrome (HUS) is the most frequent cause of acute renal failure in children. The traditional diagnostic criteria for this syndrome include hemolytic anemia with fragmented erythrocytes, thrombocytopenia, and renal failure [1]. The epidemic form of HUS occurs mostly as a result of hemorrhagic colitis. In 90% of HUS cases, infection with Shiga toxin- (Stx-) producing *Escherichia coli* (STEC) is strongly implicated [2].

Stx is divided into two main groups, referred to as Stx1 and Stx2, which share approximately 56% amino acid sequence similarity [3]. Stx1 is essentially identical to the Stx produced by *Shigella dysenteriae*. Stx2 is a more potent cause of severe HUS and central nervous system (CNS) impairment. Stx is a 71-kDa protein composed of a 32-kDa A subunit (StxA) and five 7.7-kDa B subunits (StxB). StxA has *N*-glycosidase activity that removes the adenine of the 28S RNA within the 60S ribosomal subunit, thereby rendering ribosomes inactive for protein synthesis [4]. StxB is essential for transport of StxA to the cytosol. StxB specifically binds to the cell membrane of the globotriaosylceramide (Gb3) receptor, which is present in specific mammalian host cells [5]. Stx is then internalized with Gb3 and transported toward the trans-Golgi network [6].

Stx is thought to enter the bloodstream through inflamed intestinal mucosa, then bind with low affinity to blood cells, specifically polymorphonuclear leukocytes, thereby being transported to the target organs [7]. Gb3, the functional receptor for Stx, is present on the surfaces of various cell types, including human glomerular endothelial cells, glomerular epithelial cells [8], and human brain endothelial cells (HBECs) [9]. Stx binding to target cells expressing Gb3 inhibits protein synthesis and induces apoptosis and necrosis [10].

Approximately, 30% of patients with HUS suffer from CNS complications, and these patients have the poorest prognosis [11]. However, the pathogenesis of CNS dysfunction is not fully understood. Although thrombotic microangiopathic damage is characteristic of HUS and is thought to result from the direct cytotoxic effects of circulating Stx on the vascular endothelium [12], there is no consensus on the pathogenic mechanisms responsible for brain involvement in HUS. However, a possible role for the inflammatory response provoked by cytokines has been suggested. Clinical studies have demonstrated that patients with HUS have elevated plasma levels of cytokines such as tumor necrosis factor *α* (TNF-*α*), interleukin-1*β* (IL-1*β*), and interleukin-6 (IL-6) [13]. In the presence of TNF-*α* or IL-1*β*, the cytotoxicity of Stx towards endothelial cells is significantly increased. Furthermore, TNF-*α* and IL-1*β* upregulate the expression of Gb3 in endothelial cells [14]. Similarly, although it has been demonstrated that HBECs are very resistant to Stx-induced cytotoxicity [9], inflammatory mediators, such as TNF-*α* and/or IL-1*β*, markedly increase the sensitivity of HBECs to Stx cytotoxicity [15], which is due to modulation of expression of Gb3.

Several components of the Gb3 metabolic pathway may be involved in cytokine-stimulated Gb3 expression. Gb3 is synthesized from lactosylceramide and UDP-galactose by Gb3 synthase (GalT6) [16]. Consequently, increased Gb3 content could be due to increased activity of GalT6. Eisenhauer et al. have shown that TNF-*α* and IL-1*β* increase GalT6 activity and mRNA levels [17].

The inflammation associated with HUS is marked by the release of chemokines, and the levels of IL-8 and MCP-1 are significantly increased in urine samples collected from HUS patients [18]. Purified Stxs directly induce the expression of the neutrophil chemoattractant IL-8 in human intestinal epithelial cells [19]. These Stx-induced increases in IL-8 synthesis by intestinal epithelial cells could be important in augmenting the host mucosal inflammatory responses to STEC infection [20]. Endothelial cells exposed to Stx2 release inflammatory chemokines, such as IL-8 and MCP-1, which stimulate adhesion and transmigration of leukocytes [21]. Such chemokines may also play a role in the pathogenesis of HUS. However, in brain parenchymal cells and neuroglial cells, expression of chemokines in response to Stx has not been studied.

Several studies support the hypothesis that Stx causes brain injury in HUS. Intravenous inoculation of rabbits with Stx2 caused severe CNS injury associated with the invasion of Stx2 through the BBB [22]. Some authors have reported that intracerebroventricular administration of Stx2 causes neuronal death and glial cell damage in the rat brain. Transmission electron microscopy studies have revealed apoptotic neurons, ultrastructural alterations of glia, and demyelinated fibers [23]. In addition, confocal microscopy has demonstrated reactive astrocytes in contact with Stx2-containing neurons [24]. These findings support the contention that Stx2 has a direct effect on brain neuroglial cells.

The brain contains neuronal, glial (e.g., astrocytes, microglia, and oligodendrocytes), and endothelial cells. Human astrocytes (HASTs) are the most abundant type of glial cell in the human brain. They play key roles in development, homeostasis, inflammatory responses, and repair of the CNS by producing a wide variety of cytokines, chemokines, and growth factors and by expressing receptors for these molecules [25]. These chemokines play important roles in development, growth, cell migration, production of free radicals, apoptosis, T-cell activation, neoplasia, inflammatory regulation in response to injury, wound healing, tissue repair, and macrophage recruitment, as well as in interactions with pathogens, including viruses [26–29]. Chemokines and their receptors play important roles in several neurodegenerative and inflammatory diseases of the CNS, including trauma, stroke, Alzheimer's disease, multiple sclerosis, CNS tumors, and human immunodeficiency virus encephalitis [30–32]. We speculated that Stxs would act directly on astrocytes by inducing the production of chemokines and play a role in CNS complications. 

In the present study, we investigated how Gb3 expression, including GalT6, in HASTs is controlled by inflammatory cytokines. In addition, we sought to obtain further evidence that Stx2 acts to produce chemokines in HASTs and that these chemokines participate in the pathogenesis of CNS complications associated with HUS.

## 2. Methods

### 2.1. Reagents and Toxins

Purified Stx2 was purchased from Toxin Technology Inc. (Sarasota, FL, USA). Toxicity was determined to be greater than 10^7^ units per milligram using a Vero cell assay. All reagents were purchased from Sigma Chemical Co. (St. Louis, MO, USA).

### 2.2. Cells and Culture Conditions

HASTs derived from human fetal brain tissue (Lonza, Walkersville, MD, USA) were routinely subcultured every 6 days in Clonetics Astrocyte Growth Medium (Lonza). Cells were reseeded after reaching confluence at 3,500 cells per square centimeter and were incubated at 37°C in a humidified atmosphere containing 5% CO_2_. The HASTs in the experiments were used after two to four passages.

### 2.3. Analysis of Gb3 Expression Stimulated by IL-1*β*

Analysis of Gb3 expression stimulated by IL-1*β* was conducted by immunohistochemistry using the Vectastain Elite ABC kit (Vector Labs, Burlingame, CA, USA). HASTs were cultured with 10^−6^ mol/L IL-1*β* or with medium alone. The cells were stimulated for 48 h with IL-1*β* and fixed in 4% paraformaldehyde for 10 min at room temperature. Slides were prepared and incubated in rat anti-human CD77 IgM antibody (1 : 100 dilution; Beckman Coulter, Brea, CA, USA) or PBS for 8 h at room temperature, then washed and incubated with a 1 : 100 dilution of secondary antibody (supplied in the Vectastain kit) at room temperature for 1 h. The colorimetric reaction was obtained using 0.5 mg/mL diaminobenzidine (DAB) substrate solution (Vector Labs) with 0.02% H_2_O_2_. The slides were counterstained with Gill's hematoxylin.

### 2.4. Real-Time Quantitative PCR of GalT-6 mRNA

HASTs grown in six-well plates were incubated in IL-1*β* (10^−8^ to 10^−5^ mol/L) or TNF-*α* (10^−8^ to 10^−5^ mol/L). At 0, 8, and 24 h postinoculation, total RNA was isolated using the RNeasy Mini kit (Qiagen, Valencia, CA, USA), with an RNase-free DNase (Qiagen) treatment for 30 min in accordance with the manufacturer's instructions. RNA was reverse transcribed to produce cDNA using the Prime Script RT reagent kit (Takara Bio, Otsu, Japan). Real-time quantitative polymerase chain reaction (PCR) was performed using SYBR Green PCR Master Mix and the Thermal Cycler Dice Real-Time System (Takara Bio). All experiments were carried out five times. The real-time PCR primers specific for GalT-6 and the housekeeping gene glyceraldehyde-3-phosphate dehydrogenase (GAPDH) are listed in Table 1. The PCR conditions were 95°C for 10 s, followed by 40 cycles of 95°C for 5 s and 60°C for 30 s. The relative amounts of mRNA were determined from the threshold cycle values of the reference gene, GAPDH. All further calculations and statistical analyses were carried out based on these values and are referred to as relative expression ratios.

### 2.5. Chemokine Enzyme-Linked Immunosorbent Assay (ELISA)

HASTs grown in six-well plates were incubated in 10^−9^ and 10^−10^ mol/L Stx2. At 0, 24, and 48 h postinoculation, cell supernatants from treated HASTs were collected by centrifugation and stored at −80°C until further analysis. The ELISAs for IL-8 and monocyte chemoattractant protein-1 (MCP-1) were performed using the corresponding Quantikine Colorimetric Sandwich ELISA kits (R&D Systems, Minneapolis, MN, USA) in accordance with the manufacturer's protocol. Chemokines were quantified by measurement of absorbance at 450 nm (*A*_450_) with a 680 Microtiter Plate Reader (Bio-Rad Laboratories, Hercules, CA, USA). Chemokine protein concentrations were calculated based on the standard curves. The assay sensitivities were 4 pg/mL (IL-8) and 5 pg/mL (MCP-1).

### 2.6. Real-Time Quantitative PCR of Chemokine mRNA

HASTs grown in six-well plates were incubated with either 10^−9^ or 10^−10^ mol/L Stx2, 10^−9^ mol/L Stx2 with 10^−6^ mol/L IL-1*β*, or 10^−6^ mol/L IL-1*β* alone. At 0, 8, and 24 h postinoculation, total RNA was extracted and real-time quantitative PCR performed using the methods described previously. The real-time PCR primers specific for IL-8 and MCP-1 (Takara Bio) are listed in Table 1. The PCR conditions were one cycle at 95°C for 10 s, followed by 40 cycles of 95°C for 5 s and 60°C for 30 s. The relative amounts of the mRNA were determined as described previously.

### 2.7. Statistics

The means ± SD were determined, and the mean values in the two separate groups were compared using a Student's *t*-test. Differences at *P* < 0.05 were considered to be significant.

## 3. Results

### 3.1. Immunohistochemical Analysis of Gb3 Expression in HASTs Treated with IL-1*β*

**Figure 1:**
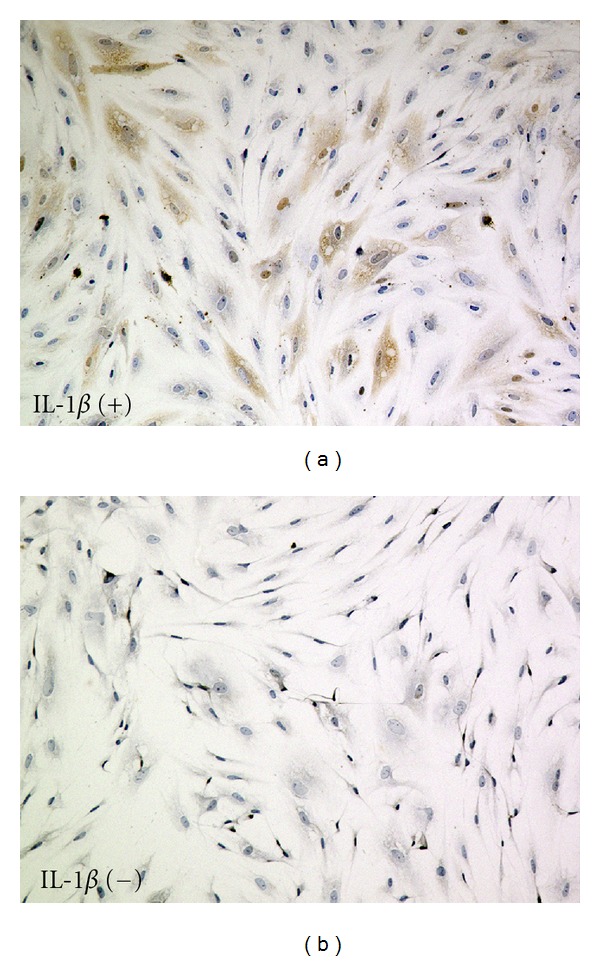
Immunohistochemical analysis of Gb3 expression in HASTs. Immunohistochemistry was performed on HASTs treated with IL-1*β*. A condensed brown DAB precipitate was observed in HASTs. A relatively high level of Gb3 was detected in HASTs treated with 10^−6^ mol/L IL-1*β* for 48 h (a), compared with the negative control (b) (original magnification ×100).

Proinflammatory cytokines increase the expression of Stx receptor Gb3 on the surface of different Stx target cells. To determine the levels of Gb3 expression in HASTs treated with IL-1*β*, immunohistochemistry was performed. In HASTs treated for 48 h with 10^−6^ mol/L IL-1*β*, a relatively high level of Gb3 (Figure 1(a)) was detected in comparison to the untreated controls (Figure 1(b)). Conversely, no change in Gb3 level was detected in HASTs treated with TNF-*α* (data were not shown).

### 3.2. Real-Time Quantitative PCR Analysis of GalT6 mRNA Induction in HASTs Treated with IL-1*β* or TNF-*α*

**Figure 2:**
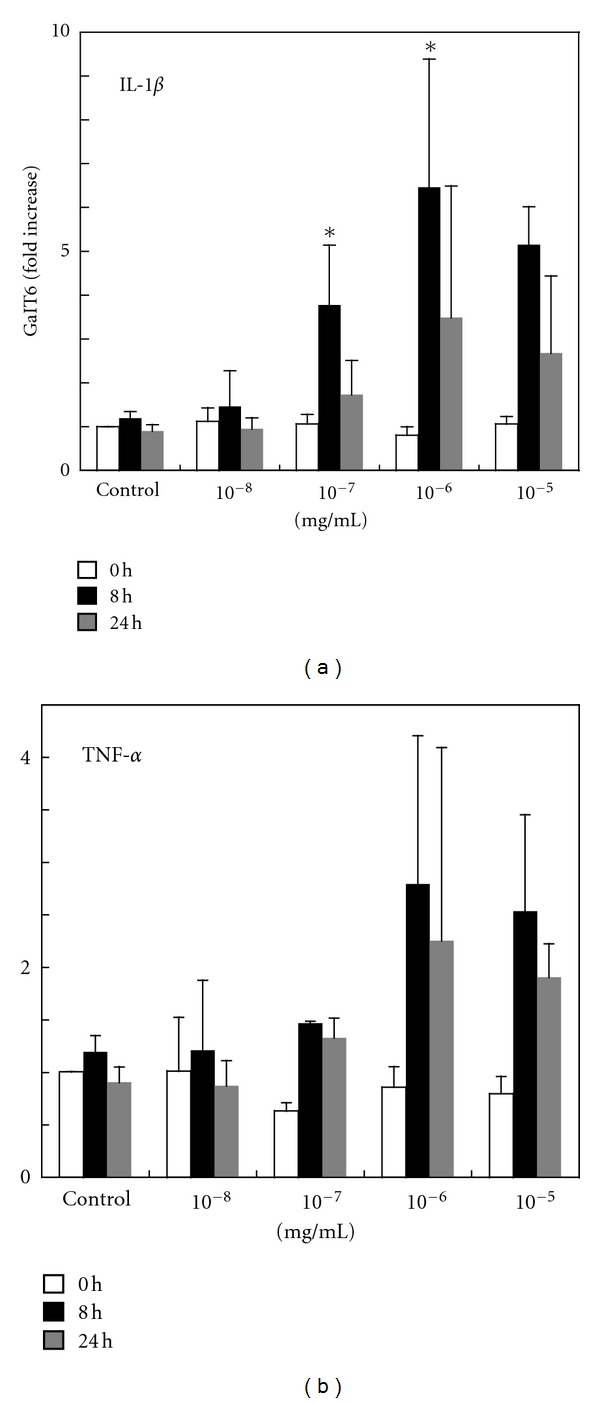
Real-time quantitative PCR analysis of GalT6 mRNA induction in HASTs treated with IL-1*β* and TNF-*α*. Expression of GalT6 mRNA was examined by real-time quantitative PCR using HASTs treated with IL-1*β* (10^−8^ to 10^−5^ mol/L) (a) and TNF-*α* (10^−8^ to 10^−5^ mol/L) (b) for 8 or 24 h. The levels of induction of GalT6 mRNA expression were calculated relative to the control cultures. Data are expressed as the fold increase of mRNA (mean ± SD; SD values from five independent experiments). Comparison of GalT6 mRNA levels elicited by treatment with IL-1*β* or TNF-*α* with those of the untreated control. *P < 0.05.

It has been demonstrated using HBECs and intestinal epithelial cells that inflammatory cytokines increase Gb3 expression levels by generating lactosylceramide through the activation of GalT6. Immunohistochemistry revealed that the expression of Gb3 in HASTs was increased by IL-1*β*. In addition, we investigated the expression of GalT6 mRNA relative to that of the untreated controls following treatment with IL-1*β* (10^−8^ to 10^−5^ mol/L) or TNF-*α* (10^−8^ to 10^−5^ mol/L) for 8 or 24 h. At 8 h, IL-1*β* (10^−7^ to 10^−5^ mol/L) treatment resulted in significant induction (*P* < 0.05) of GalT6 mRNA relative to the untreated controls (Figure 2(a)). A 6.5-fold increase in the level of GalT6 mRNA was induced with 10^−6^ mol/L IL-1*β*. TNF-*α* treatment showed a similar trend to IL-1*β* treatment at 8 h, but it was not significant (Figure 2(b)).

### 3.3. Expression of Chemokine mRNA in HASTs upon Stimulation with Stx2

**Figure 3:**
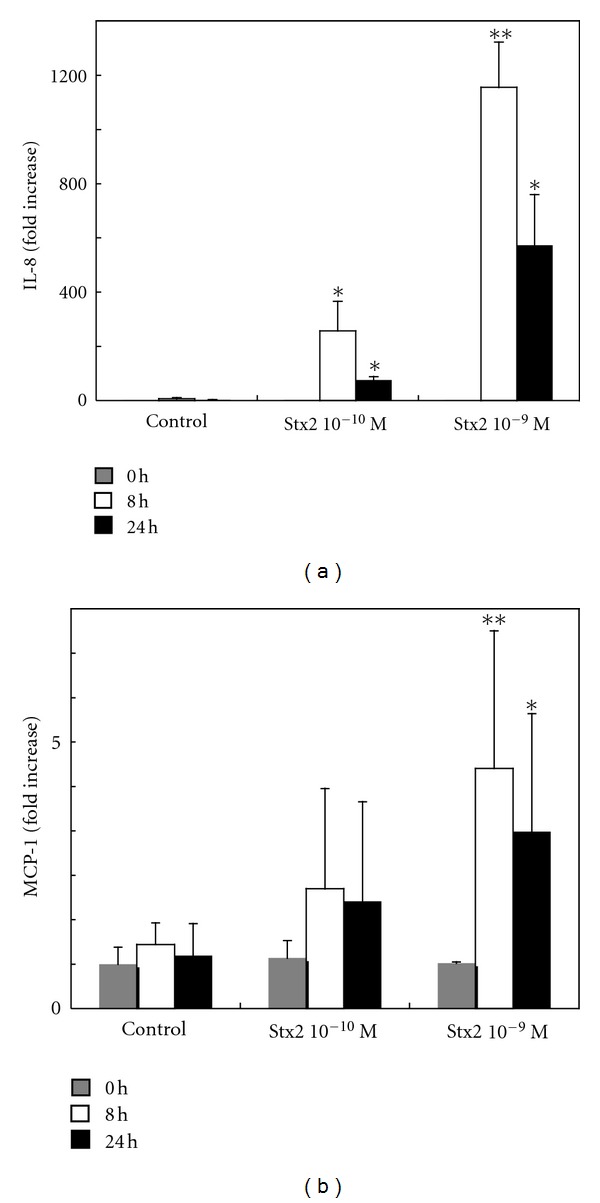
Expression of chemokine mRNA in HASTs upon stimulation with Stx2. The expressions of IL-8 (a) and MCP-1 (b) mRNA was examined by real-time quantitative PCR using HASTs treated with 10^−10^ to 10^−9^ mol/L Stx2 for 8 or 24 h. The levels of induction of IL-8 and MCP-1 mRNA were calculated relative to the control cultures. Data are expressed as fold increases of mRNA (mean ± SD from five independent experiments). Comparisons were made between the levels of IL-8 and MCP-1 mRNA elicited by Stx2 and those of the untreated controls. *P < 0.05 and **P < 0.01.

Astrocytes are an important source of chemokines that play a role in brain inflammatory responses. To investigate whether expression of IL-8 and MCP-1 mRNA was increased in HASTs treated with Stx2, we used real-time PCR. Following treatment with 10^−10^ to 10^−9^ mol/L Stx2 for 8 or 24 h, real-time quantitative PCR was performed to verify the expression of IL-8 and MCP-1 transcripts. Following treatment with Stx2, IL-8 mRNA induction was significantly elevated (*P* < 0.05) relative to the untreated controls (Figure 3(a)). A 256-fold increase in the level of IL-8 mRNA was induced by 10^−10^ mol/L Stx2, and a 1,155-fold increase was induced with 10^−9^ mol/L Stx2 at 8 h. A 5.7-fold increase of MCP-1 mRNA was induced by 10^−10^ mol/L Stx2, and an 11.6-fold increase was induced with 10^−9^ mol/L Stx2 at 8 h, in comparison with the untreated controls (Figure 3(b)). 

### 3.4. Chemokine Release by HASTs upon Stimulation with Stx2

**Figure 4:**
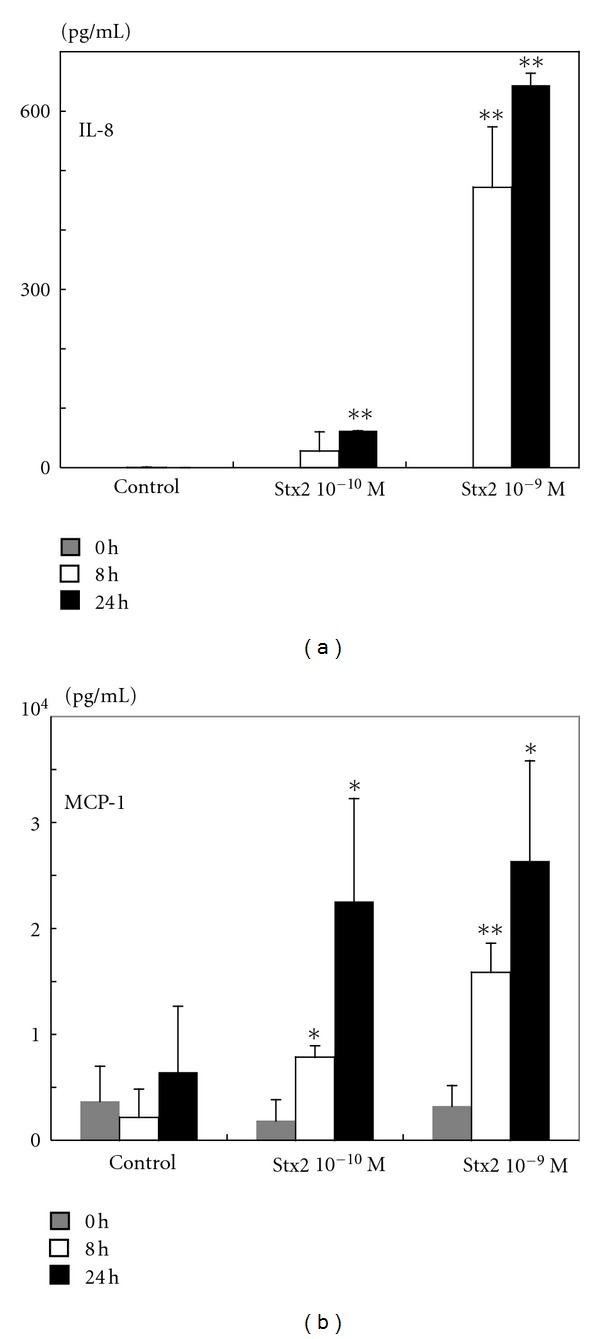
Chemokine release by HASTs upon stimulation with Stx2. Secretion of IL-8 (a) and MCP-1 (b) by HASTs treated with 10^−9^ or 10^−10^ mol/L Stx2 for 24 or 48 h. IL-8 and MCP-1 were measured in cell culture supernatants using ELISA. Data are the means ± SD from the five experiments. Levels of Stx2-induced IL-8 or MCP-1 and those in the untreated controls were calculated. *P < 0.05 and **P < 0.01.

To determine whether the upregulation of chemokine mRNA was accompanied by release of these chemokines into the medium, HASTs were exposed to 10^−9^ mol/L and 10^−10^ mol/L Stx2 for 24 and 48 h, and the concentrations of chemokines in the medium were measured by ELISA. Treatment of HASTs with 10^−9^ mol/L and 10^−10^ mol/L Stx2 increased secretion of IL-8 into the medium relative to the control cultures at 24 and 48 h (Figure 4(a)). The concentrations of IL-8 in the medium were significantly higher in the Stx2-treated cultures, especially for 10^−9^ mol/L Stx2, than those in the control cultures at 24 and 48 h. Similarly, treatment of HASTs with 10^−9^ mol/L and 10^−10^ mol/L Stx2 increased the secretion of MCP-1 into the medium relative to the control cultures at 24 and 48 h (Figure 4(b)). Unlike IL-8, MCP-1 was released in the absence of Stx2 stimulation (Figure 4(b)). 

### 3.5. Expression of IL-8 mRNA in HASTs upon Stimulation with Stx2 and IL-1*β*

**Figure 5:**
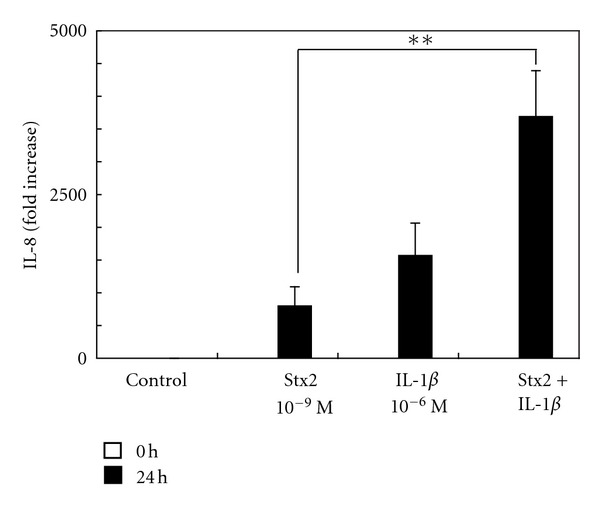
Expression of IL-8 mRNA in HASTs upon stimulation with Stx2 and IL-1*β*. Expression of IL-8 mRNA was examined by real-time quantitative PCR using HASTs treated with 10^−9^ mol/L Stx2 and 10^−6^ mol/L IL-1*β* for 24 h. The levels of induction of IL-8 mRNA expression were calculated relative to those of the control cultures, which had not been treated with Stx2 or IL-1*β*. Data are expressed as fold increases of mRNA expression (mean ± SD from five independent experiments). Comparisons between the levels of IL-8 mRNA elicited by treatment with 10^−9^ mol/L Stx2 alone and those elicited with 10^−9^ mol/L Stx2 and 10^−6^ mol/L IL-1*β* are shown. Induction of IL-8 mRNA upon stimulation with 10^−9^ mol/L Stx2 and 10^−6^ mol/L IL-1*β* in combination at 24 h was significantly elevated (*P* < 0.01) in comparison with that elicited by either 10^−9^ mol/L Stx2 or 10^−6^ mol/L IL-1*β* alone. **P < 0.01.

Using real-time PCR, we found that the expression of GalT6 mRNA was increased by stimulation with IL-1*β*, and immunohistochemistry demonstrated that the level of Gb3 expression in HASTs was also increased by IL-1*β* stimulation. To investigate whether the increase of Gb3 expression induced by IL-1*β* stimulation correlated with the expression of chemokine mRNA, we treated HASTs with Stx2 and IL-1*β*. Induction of IL-8 mRNA upon stimulation with 10^−9^ mol/L Stx2 and 10^−6^ mol/L IL-1*β* combined was significantly greater (*P* < 0.05) at 24 h than that resulting from stimulation with either agent alone at the same concentration (Figure 5). A 3,700-fold increase was induced by this combination of Stx2 and IL-1*β*, in comparison with the control levels. Coincubation of Stx2 with IL-1*β* did not elicit a significant increase in the level of MCP-1 mRNA (data were not shown).

## 4. Discussion

CNS dysfunction is an important determinant of prognosis and mortality in children with HUS [11]. Although the pathogenesis of CNS involvement in HUS is not fully understood, the disruption and increased permeability of the BBB and neuronal disturbance resulting from HBEC injury are central events in CNS complications observed during the acute phase of HUS [15].

In a previous histopathologic study, Mizuguchi et al. showed that intravenous administration of Stx2 to rabbits resulted in hemorrhage and necrosis of both neural and endothelial cells in the brain [33]. Moreover, an *in vivo* study has shown that Stx2 injected intravenously into rabbits was detected in the cerebrospinal fluid (CSF) and that influx of Stx2 into the CSF caused injury to the tight junctions between ependymal cells, resulting in destruction of the BBB [16]. Some authors have reported that intracerebroventricular administration of Stx2 causes neuronal death and glial cell damage in the rat brain [23]. However, it is unclear how Stx2 affects neuroglial cells during an inflammatory response. 

HASTs comprise 55%–60% of the total number of human brain cells [34] and are involved in virtually every type of brain pathology. HASTs play key roles in the development, inflammation, and repair of the CNS by producing a wide variety of chemokines. Under inflammatory or otherwise pathologic conditions, HASTs become activated and display enhanced production of several cytokines, chemokines, and growth factors. In addition, HASTs generally start to proliferate under such conditions, a phenomenon known as astrogliosis. Therefore, knowledge of the cellular response of HASTs to Stx is essential to understand the neuropathology observed in the severe cases of HUS.

In this study, IL-1*β* treatment of HASTs increased both Gb3 and GalT6 mRNA expression, as determined by immunohistochemistry and real-time PCR, respectively. In addition, treatment of HASTs with Stx2 induced production of the chemokines IL-8 and MCP-1. A mixture of Stx2 and IL-1*β* induced IL-8 to a marked degree. These findings suggest that Stx2 triggers an immune response through the expression of chemokines in HASTs.

Toxin binding to Gb3 is the primary determinant of the cytotoxic and pathologic effects of Stx protein. Previous studies have highlighted the role of proinflammatory agents in enhancing the sensitivity of different target cells, including HBECs, to the effects of Stx through upregulation of Gb3 expression [9, 35]. Gb3-expressing cells are recognized to be the targets of Stx. Primary HBECs become susceptible to Stx when they express Gb3 in response to TNF-*α*, further exacerbating the disease process [15]. Therefore, we investigated whether Gb3 expression in HASTs is increased by inflammatory cytokines. Using real-time PCR, we determined that the expression of GalT6 mRNA was increased by IL-1*β* stimulation, and immunohistochemistry showed that the level of Gb3 expression in HASTs was increased by IL-1*β* stimulation. These results demonstrated that HASTs, which are CNS parenchymal cells, express Gb3, and that this expression is increased by inflammatory cytokines. In HBECs, IL-1*β* and TNF-*α* increased the expression of Gb3 and enhanced the toxicity of Stx. Only IL-1*β* increased the expression of GalT6 mRNA and increased the level of Gb3 expression in HASTs. Treatment with TNF-*α* elicited a response similar to that for IL-1*β* in terms of the expression of GalT6 mRNA at 8 h, although the degree of similarity was not significant. In this study, the mechanism by which IL-1*β* increased the level of Gb3 expression in HASTs more than TNF-*α* is not understood, although it may be a result of differences in cell types, and further investigations of this are required. 

Chemokines, a family of proinflammatory cytokines, are divided into two subfamilies: the CXC family, represented in this study by IL-8, and the CC family, represented here by MCP-1. Chemokines stimulate target cell-specific directional migration of leukocytes and may be potent mediators of inflammatory processes. Human IL-8 is a proinflammatory chemokine secreted predominantly by monocytes, endothelial cells, and glial cells and acts as a chemoattractant and activator of neutrophils via CXCR1 and CXCR2 receptor signaling. MCP-1 is a potent chemotactic factor for monocytes and plays an important role in the regulation of repair processes and cellular interactions in the CNS [25, 26]. It can be expressed by many cell types including microglia, astrocytes, and neurons [20, 21].

In recent years, it has been shown that chemokines play many roles in inflammation in the CNS and are responsible for the development of various neurodegenerative diseases and ischemic damage to brain cells [17, 18]. MCP-1 and IL-8 are upregulated in the brains of patients with Alzheimer's disease [24]. In addition, the levels of MCP-1 and IL-8 are significantly elevated in the CSF of infants with amyotrophic lateral sclerosis, Japanese encephalitis, or hypoxia-ischemia [36].

We found that by stimulating HASTs with Stx2 chemokines such as IL-8 and MCP-1 were produced, the production of IL-8 being particularly marked. Elevated levels of IL-8 and MCP-1 mRNA were found in toxin-treated cells in comparison to the untreated controls. IL-8 and MCP-1 proteins were found at higher levels in the conditioned media of Stx2-treated cells than in those of untreated cells, and the chemokines IL-8 and MCP-1 were produced by HASTs upon stimulation with Stx. Several studies have demonstrated that Stx induces the expression of IL-8 and MCP-1 in intestinal epithelial cells, endothelial cells, and monocytes and that the levels of IL-8 and MCP-1 are significantly increased in urine samples collected from patients with HUS. It has, therefore, been suggested that chemokines such as IL-8 and MCP-1 play a specific role in the pathogenesis of HUS. For HUS patients, a high peripheral polymorphonuclear neutrophil count at presentation was strongly associated with a poor prognosis [37]. Activated polymorphonuclear neutrophils could contribute to the brain inflammatory response and participate in the secondary damage following BBB disruption in HUS. Stx2 induces expression of several chemokines, such as IL-8 and MCP-1, in astrocytes, and might constitutively induce the inflammatory response. In a mouse model of HUS, neutralization of chemokines resulted in decreased renal fibrin deposition, suggesting that disrupting or eliminating these macrophage chemokines may be a useful treatment for the kidney damage associated with HUS [38]. Currently, it is unclear whether IL-8 and MCP-1 are induced in the CNS. In the present study, we found that astrocytes were responsive to Stx2 and produced IL-8 and MCP-1. In biological systems, further studies will need to address whether IL-8 and MCP-1 are induced by Stx2 in the CNS, and what effects they exert. 

No therapy for HUS encephalopathy has yet been established. However, Fujii et al. showed that betamethasone sodium phosphate pulse therapy reduced the mortality rate and prolonged the survival period of rabbits with Stx2-toxemia [39]. Betamethasone sodium phosphate is routinely used as a steroid therapy for patients with brain edema. Dexamethasone reduces the permeability of the normal BBB in mice [40]. In brain astrocytes, Kim et al. showed the coordinated regulation of angiotensin-1 and vascular endothelial growth factor (VEGF) by dexamethasone, suggesting a novel mechanism underlying glucocorticoid-induced stabilization of the BBB [41]. In addition, Rock et al. examined cytokine and chemokine expression with and without dexamethasone in human microglia and astrocytes infected with *Mycobacterium tuberculosis*. They reported that treatment with dexamethasone effectively suppressed production of these mediators [42]. Steroid protective effects are thought to be mediated by immunomodulatory activities, such as a decrease in the production of proinflammatory cytokines. Therefore, anti-inflammatory compounds may have therapeutic potential for Stx-induced neurological manifestation. Investigations into the glucocorticoid regulation of chemokine expression by Stx2 in HASTs are currently underway.

In summary, our results suggest a possible relationship between excessive chemokine production in the CNS and brain damage once Stx2 reaches the brain parenchyma. We propose that suppression of chemokine production could be a new strategy for treatment of CNS damage in HUS encephalopathy. 

## 5. Conclusions

In this study, we investigated the inflammatory responses in human astrocytes treated with Stx2. Immunohistochemistry and real-time PCR revealed that the expression of globotriaosylceramide (Gb3), the receptor for Stx2, and Gb3 synthase (GalT6) in HASTs was increased by interleukin-1*β* (IL-1*β*). Chemokine mRNA expression in astrocytes was significantly upregulated by Stx2. Chemokines in the CNS may be associated with the pathogenesis of CNS manifestations with HUS.

## Figures and Tables

**Table 1:** Primer sequences.

Gene	Primer (forward)	Primer (reverse)
GalT6	ACCTGCGGAACCTGACCAAC	CATGCACAGCGCCATGAAC
IL-8	ACACTGCGCCAACACAGAAATTA	TTTGCTTGAAGTTTCACTGGCATC
MCP-1	GCTCATAGCAGCCACCTTCATTC	GGACACTTGCTGCTGGTGATTC
GAPDH	GCACCGTCAAGGCTGAGAAC	TGGTGAAGACGCAGTGGA
